# Reduction of the domino effect in osteoporotic vertebral compression fractures through short-segment fixation with intravertebral expandable pillars compared to percutaneous kyphoplasty: a case control study

**DOI:** 10.1186/1471-2474-14-75

**Published:** 2013-03-02

**Authors:** Jui-Yang Hsieh, Chung-Ding Wu, Ting-Ming Wang, Hsuan-Yu Chen, Chui-Jia Farn, Po-Quang Chen

**Affiliations:** 1 Department of Orthopedics, National Taiwan University & Hospital, No. 7, Chung-Shan South Rd., 100, Taipei, Taiwan; 2 Department of Orthopaedics, Min-Sheng General Hospital, 168, Jingguo Rd., 330, Taoyuan City, Taoyuan County, Taiwan; 3 Department of Orthopaedics, Cathay General Hospital, No. 280, Sec. 4, Ren’ai Rd., Xinyi Dist., 110, Taipei City, Taiwan

**Keywords:** Short-segment fixation, Intravertebral expandable pillar, Percutaneous kyphoplasty, Vertebral compression fracture

## Abstract

**Background:**

Osteoporotic vertebral compression fracture is the leading cause of disability and morbidity in elderly people. Treatment of this condition remains a challenge. Osteoporotic vertebral compression fractures can be managed with various approaches, but each has limitations. In this study, we compared the clinical outcomes obtained using short-segment fixation with intravertebral expandable pillars (I-VEP) to those obtained with percutaneous kyphoplasty in patients who had suffered vertebral compression fractures.

**Methods:**

The study included 46 patients with single-level osteoporotic thoracolumbar fractures. Twenty-two patients in Group I underwent short-segment fixation with I-VEP and 24 patients in Group II underwent kyphoplasty. All patients were evaluated pre- and postoperatively using a visual analogue scale, anterior height of the fractured vertebra, and kyphotic angle of the fractured vertebra. The latter 2 radiological parameters were measured at the adjacent segments as well.

**Results:**

There was no significant difference between the groups in terms of gender or fracture level, but the mean age was greater in Group II patients (p = 0.008). At the 1-year follow-up, there were no significant differences in the visual analogue scale scores, anterior height of the fractured vertebra, or the value representing anterior height above the fractured vertebra and kyphotic angle below the fractured vertebra, after adjusting for the patients’ gender, fracture level, and age. When considered separately, the anterior height below the fractured vertebra was significantly higher and the kyphotic angle above the fractured vertebra was significantly smaller in Group I than in Group II (p = 0.029 and p = 0.008, respectively). The kyphotic angle of the fractured vertebra was significantly smaller in Group II than in Group I (p < 0.001).

**Conclusions:**

In older individuals with vertebral compression fractures, kyphoplasty restored and maintained the collapsed vertebral body with less kyphotic deformity than that induced by short-segment fixation with I-VEP. Short-segment fixation with I-VEP was more effective in maintaining the integrity of adjacent segments, which prevented the domino effect often observed in patients with osteoporotic kyphotic spines.

## Background

Osteoporotic vertebral compression fracture (VCF) is the leading cause of disability and morbidity in elderly people. This condition is associated with severe and prolonged pain that can markedly alter the individual’s participation in daily life activities. Treatment of this condition remains a challenge [1]. Percutaneous vertebroplasty can provide effective pain relief for patients with VCF. This technique stabilizes the fracture through the use of cement for mechanical augmentation [2]. When performed with an expandable balloon, percutaneous balloon kyphoplasty (KP) is more effective in restoring vertebral height and correcting (partially) sagittal alignment [3].

Although vertebroplasty and KP are relatively safe and easy procedures to perform, complications from these procedures can be problematic. Cases of pulmonary embolism, infection, bleeding, and nerve or spinal cord compression due to the leakage of polymethyl methacrylate (PMMA) have been documented [4-6]. Although the extravasation of cement is well tolerated in majority of the patients, the leakage of cement is the cause for most symptomatic complications that lead to permanent or transient damage following vertebroplasty [7]. Notably, vertebroplasty and KP may contribute to the pathogenesis of new fractures in the adjacent vertebrae. Some of the associated complications may even require revision surgery [8-11].

Posterior surgical instrumentation and fusion are the preferred techniques for managing painful VCF. However, these techniques carry substantial risks of major complications in elderly and osteoporotic patients who have undergone long-segment fixation. Long-segment fixation may also fail when the bone involved is fragile [12,13]. Recently, the I-VEP had been effective in restoring the body height of the compressed vertebra and providing proper stiffness for the collapsed vertebra in an osteoporotic patient in vitro biomechanical study [14]. The use of intravertebral expandable pillars (I-VEP) represents an alternative method for posterior short-segment fixation that may safely provide long-lasting pain relief and reduce kyphosis. To our knowledge, however, no previous report has compared the outcomes of KP and short-segment fixation with I-VEP in the treatment of collapsed vertebrae in osteoporotic patients. This study aims to compare the clinical outcomes with respect to pain relief, stabilization, height restoration in the fractured vertebra and preservation of the segments adjacent to collapsed vertebrae in osteoporotic patients.

## Methods

The data were retrospectively collected at National Taiwan University Hospital and Min-Sheng General Hospital between May 2006 and November 2010. Each patient included was indicated for surgical intervention in the thoracic or lumbar spine region. The indications reported for the patients in this study were intractable back pain due to acute or chronic VCF, pain refractory to nonsurgical treatment for more than 6 months, or bony cleft formation in the vertebral body. The contraindications were primary or metastatic lesions with vertebral fractures, an infectious origin or poor general condition with a high risk requirement of general anesthesia. This study included 46 consecutive patients with single-level osteoporotic thoracolumbar fractures. Twenty-two patients who consulted PQC were allocated to Group I and received treatment of short-segment fixation with I-VEP. Twenty-four patients who consulted another senior orthopedic surgeon (CDW) were allocated to Group II and received treatment of KP. The study was in compliance with the WMA Declaration of Helsinki. The study based exclusively on clinical records was conducted retrospectively and received institutional review board approval from National Taiwan University Hospital (#201111054RIC). The patients in this study provided written informed consent for the publication of individual data and accompanying clinical images.

**Figure 1:**
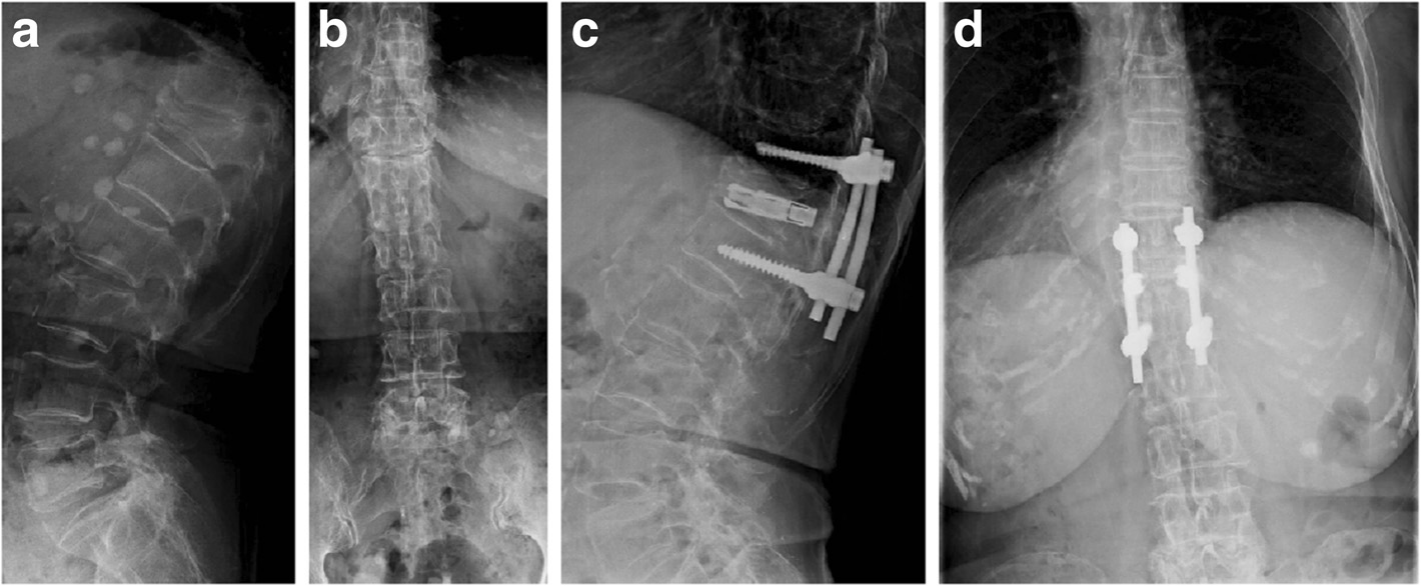
**Radiographs of Patient 10 in Group I show a T12 vertebral compression fracture before the operation and at the one-year follow-up. a** Preoperative sagittal view. **b** Preoperative anteroposterior view. **c** Postoperative lateral view. **d** Postoperative anteroposterior view.

**Figure 2:**
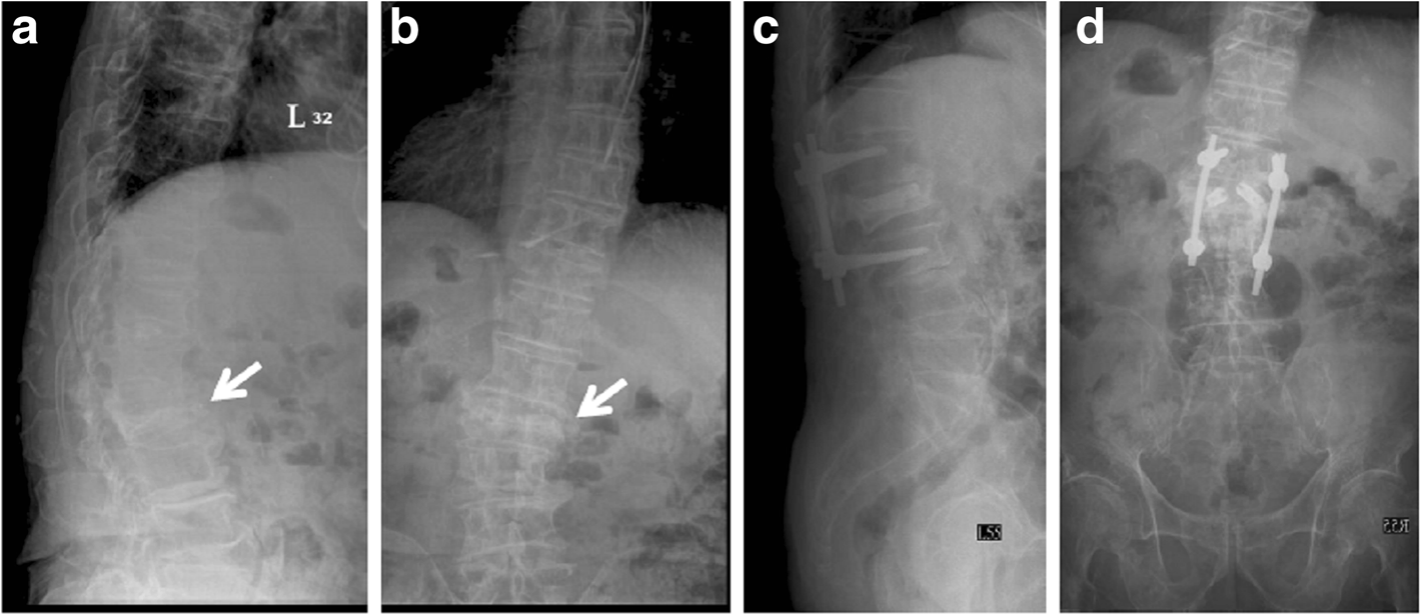
**Patient 5 in Group I is a 79-year-old man who was treated with short-segment fixation with I-VEP due to vertebral compression fracture of L2. a** Lateral-view radiograph of Patient 5 in Group I shows an L2 vertebral compression fracture before the operation. **b** Anteroposterior view of the preoperative radiograph. **c** Lateral-view radiographs at the one-year follow-up. **d** Anteroposterior view at the one-year follow-up.

**Figure 3:**
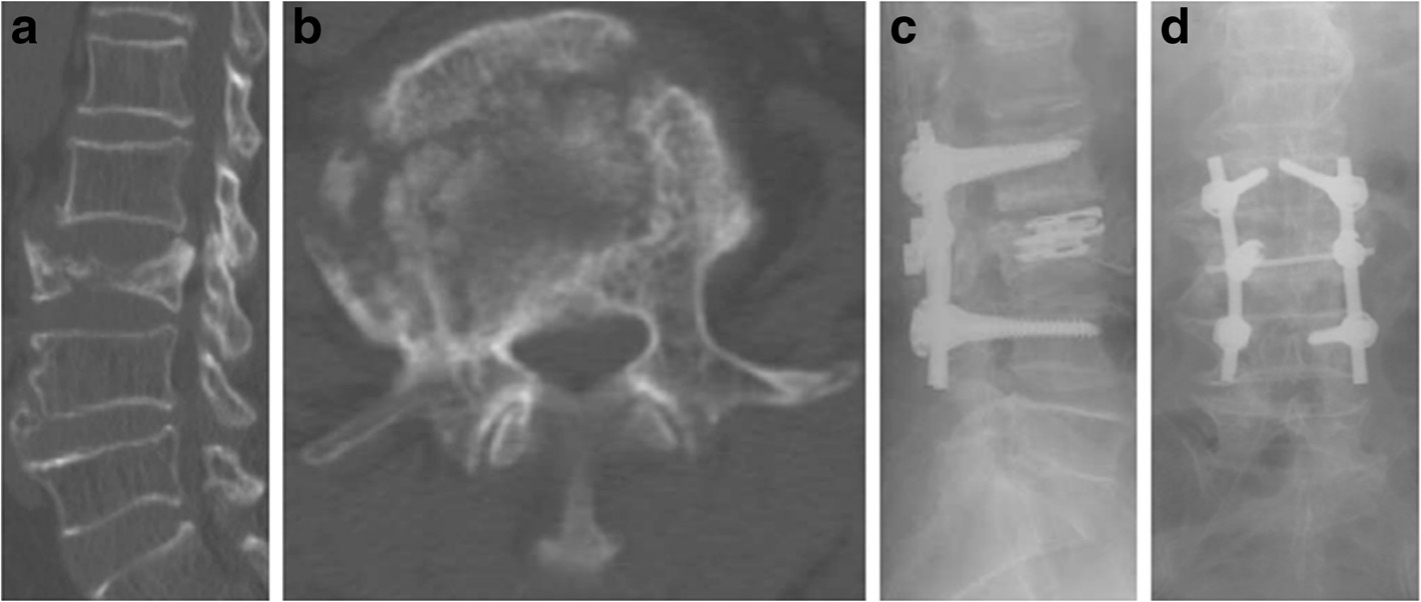
**Patient 20 in Group I (70 years old female) with an L3 concave H-shaped burst fracture underwent I-VEP insertion at L3 combined with additional short segment fixation (L2-L4). a** Preoperative CT, sagittal view. **b** Preoperative CT, axial view. **c** Lateral-view radiograph taken postoperatively. **d** Anteroposterior-view radiograph, taken postoperatively.

**Figure 4:**
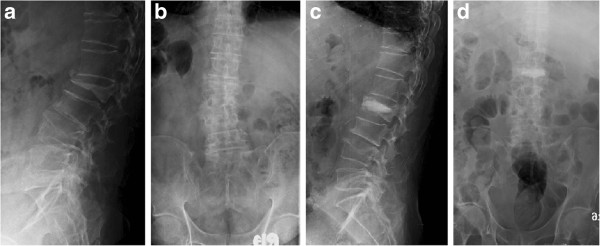
**Radiographs of Patient 15 (76 years old male) in Group II show an L2 vertebral compression fracture before the operation and at the one-year follow-up. a** Preoperative sagittal view. **b** Preoperative anteroposterior view. **c** Postoperative lateral view. **d** Postoperative anteroposterior view.

Short-segment fixation was defined as posterior stabilization enhanced by the pedicle screw and rod system (Diapason, Stryker Corp, Allendale, NJ; Aaxter Posterior Spinal System, Aaxter Co., Ltd., Taipei, Taiwan) and bone grafting one level above and one level below the injured vertebra. Abundant bone chips were packed meticulously into the void space of the collapsed vertebral body through the pedicle tract. Next, I-VEP (Aaxter Pillar Vertebral Spacer, Aaxter Co., Ltd.) that had been filled up with morcelized autologous cancellous bone chips were transpedicularly screwed into the posterior side of the vertebral body (Figures 1, 2 and 3). Bone-cement kyphoplasty (VCF-X, Bone Filler Delivery System, Central Medical Technologies, Inc., Taipei, Taiwan) was performed according to the standard balloon kyphoplasty procedure (Figure 4) [15].

**Figure 5:**
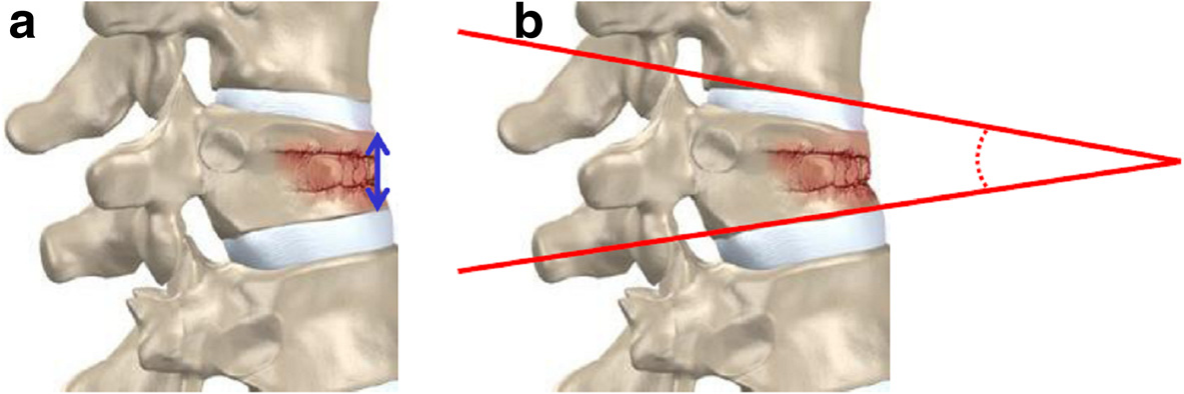
**Schematic diagrams of the radiographic measurements. a** The anterior vertebral body height of the fractured vertebra (double arrow) is the actual height of the anterior cortex of the vertebral body as measured on the lateral radiograph. **b** Measuring on a lateral radiograph with modified Cobb method requires inferior endplates above the fractured vertebra for kyphotic angle measurement.

All patients assessed their pain before and 1 year after surgery using a 10-cm visual analogue scale (VAS). Imaging using a compression ratio of the anterior height (AH) of the fractured vertebra and local kyphotic deformity angle (KA) of the fractured vertebra was performed prior to the procedure and 12 months postoperatively (Figure 5). Measurements of AH and KA of the fractured vertebra in adjacent segments were radiographically documented just above or below the fracture level despite the presence of pedicle screws. During the course of treatment, symptomatic levels of VCF between T10 and L2 were defined as Level 2; those above T9 as Level 1; those below L3 as Level 3. On postoperative day 2 or 3, all patients were encouraged to carry a cane and wear a thoracolumbar brace while walking. This protection was supposed to be maintained for 3 months. After discharge, patients were regularly followed up and evaluated after 1 week, as well as after 1, 3, 6 and 12 months on the basis of VAS pain scores and radiographs [16].

The data were evaluated using chi-square tests for gender and fracture, analysis of variance (ANOVA) for age, and analysis of covariance (ANCOVA) for the clinical outcomes. The level of statistical significance was set at p < 0.05.

## Results

The patient characteristics are presented in Tables 1 and 2. Nineteen women and 3 men in Group I and 17 women and 7 men in Group II were treated. There were no significant gender differences between the groups (p = 0.289). The number of patients’ VCF level in Group I was as follows: Level 1, 1; Level 2, 16; and Level 3, 5. The number of patients’ VCF level in Group II was as follows: Level 1, 2; Level 2, 16; and Level 3, 6.

**Table 1:** Demographic and clinical characteristics of Group I patients treated with short-segment fixation with I-VEP

**Group I**	**Gender**	**Age (y/o)**	**Level**	**VAS**	**AH (mm)**	**KA (°)**	**AH above (mm)**	**AH below (mm)**	**KA above (°)**	**KA below (°)**	**VAS**	**AH (mm)**	**KA (°)**	**AH above (mm)**	**AH below (mm)**	**KA above (°)**	**KA below (°)**
				**Pre OP**							**Post OP 1Y**						
1	F	85	L3	9	6	16	27.3	16.9	0	1,0	1	16.8	10.6	27.3	17.2	3	4
2	M	83	L4	8	21	5.7	32.7	26.5	1.5	1.9	0	26	1.2	31.2	28.3	1.7	1.3
3	F	80	L1	8	14.3	19.7	26.4	33.2	2.3	3	0	19.6	15	26.4	33.3	2	3.2
4	F	78	L2	8	3	26.2	27.8	27	1.5	3.1	1	17.7	8.1	27.8	27.1	0.2	0.7
5	M	79	L2	9	4.7	24.3	25.9	30.3	6.5	6.1	2	18.4	8.8	26.5	28.9	5.2	2.2
6	F	79	T12	8	10.3	26.5	26.6	25	8.2	12.3	3	14.6	11.9	25.3	26	7.1	8.2
7	F	67	T12	9	6.7	30	22	27.6	5.8	6.4	3	15.2	3.9	22.5	27	5.3	5.8
8	F	60	L3	8	15.8	20.3	30	32	5.5	2.5	5	19.5	9.6	30	32.3	5.4	2.5
9	F	70	L2	10	2.81	17.2	24.9	26.1	0.12	0.51	1	20	14	25.1	26	0.1	0.5
10	F	74	T12	10	12.6	24.1	25.8	30.3	3.2	2.5	1	19.6	19.2	25.2	30.4	3.7	2.3
11	F	76	T8	9	18.3	1.5	24	24	5.2	0.3	2	10.4	15.7	23.7	23.8	5.5	0.5
12	F	76	L2	10	13.8	12.9	24.6	21.3	9.7	0.9	1	21	12.2	24.7	21.1	9.5	0.9
13	F	69	L2	9	14.9	25.7	27.2	29.5	1.5	2.3	1	16.9	16.6	26.9	29.4	1.4	2.3
14	F	69	L4	9	16.3	13	27.3	28.2	0.15	1.8	4	20.64	3.73	27.5	28	0.1	1.9
15	F	74	T12	9	5.6	29	18.6	20.6	6	3.1	0	20.3	5.2	18.8	20.5	6.3	3
16	F	60	L1	9	14.8	19.8	29.6	30.4	9.2	8.3	0	20.59	16.9	30	30.3	9.5	8.5
17	F	71	L2	9	21.8	5.7	26	24	0.12	9.3	1	26.7	4.5	26.1	23.8	0.1	9.4
18	F	67	T21	9	13.7	12.2	30.2	24.6	2.3	2	0	18.1	9.6	30.1	24.7	2.2	2.1
19	F	76	T12	8	2.2	34.8	5.1	4.8	27.1	30.7	2	17.4	13	4.9	4.9	27.3	32
20	F	70	L3	8	20.8	9.9	29.7	28.7	1.4	1.1	0	28	1.2	27.7	31	3	0.7
21	M	80	L1	9	20.7	10.5	28.8	28.4	6.3	8.2	2	22.4	4.8	28.9	28.5	6.6	8
22	F	76	L1	10	13.6	14.7	28.4	34.3	1.1	0	2	20.8	7.1	28.2	33.9	1.2	0.2
Minima		60		8	2.2	1.5	5.1	4.8	0	0	0	10.4	1.2	4.9	4.9	0.1	0.2
Maxima		85		10	21.8	34.8	32.7	34.3	27.1	30.7	5	28	19.2	31.2	33.9	27.3	32
Mean		73.6		8.9	12.44	18.17	25.86	26.08	4.76	5.11	1.5	19.57	9.67	25.67	26.2	4.84	4.55
SD		6.5		0.7	6.22	8.61	5.4	6.18	5.73	6.65	1.3	3.89	5.18	5.29	6.2	5.67	6.61

*VAS* = visual analogue scale; *AH* = anterior height of the fractured vertebra; *KA* = kyphotic angle of the fractured vertebra; *AH* above = anterior height above the fractured vertebra; *AH* below = anterior height below the fractured vertebra; *SD* = standard deviation.

**Table 2:** Demographic and clinical characteristics of Group II patients treated with kyphoplasty

**Group II**	**Gender**	**Age (y/o)**	**Level**	**VAS**	**AH (mm)**	**KA (°)**	**AH above (mm)**	**AH below (mm)**	**KA above (°)**	**KA below (°)**	**VAS**	**AH (mm)**	**KA (°)**	**AH above (mm)**	**AH below (mm)**	**KA above (°)**	**KA below (°)**
				**Pre OP**							**Post OP 1Y**						
1	F	84	T12	8	16.08	20.3	24.9	26.4	2.02	1.01	3	16.76	0.89	9.8	27.2	12.3	3.2
2	M	79	L1	7.5	11	19.4	25	26.7	4.2	6.2	2.5	20.74	0.91	25.2	26.2	4.4	6.6
3	M	80	T12	8	6.32	21.23	24.8	27.5	10.8	12.21	2	19.96	8.5	20.4	27.1	11	9
4	F	84	L1	8	11.16	25	27	29.2	2.5	2.1	3	23.54	1.26	27.1	29.1	2.4	2.2
5	M	74	T10	6	8.61	18.6	14.4	25.7	6.8	4.3	1	15.62	1.8	15	25.6	7.6	4.4
6	F	85	T12	8	17.2	20.02	23.6	28.6	7.2	1.8	2	20.2	9.53	19.3	24.4	7.6	6.9
7	F	84	T12	8	11.4	24.41	23.5	25.3	5.8	6.2	2.5	16.63	9.19	23.2	25.3	5.5	6.3
8	F	85	L3	7.5	8.16	19	16.9	28.1	16.1	0	2.5	35.62	0.68	14.3	25.5	14.1	2
9	F	73	L1	8	16.85	16.9	25.8	29.8	4.6	0.2	1.5	14.4	0.27	25.7	29.8	4.5	0.3
10	M	76	L3	8.5	31.02	21	18	33.7	15.9	4.3	2	32.47	2.14	16.8	33.3	18	4.4
11	F	71	L3	8	33.33	18.3	28.1	21.9	0.45	11.73	1.5	33.64	0.89	23.3	22	3.2	9.8
12	M	88	L1	7	12.19	19.58	25.8	32.7	8.1	1.9	2.5	15.08	1.42	25.7	32.7	8.2	1.8
13	M	79	T12	8.5	10.85	23.82	25.2	29.1	7.7	4.7	1.5	20.82	2.01	25.3	29	7.5	4.9
14	F	56	L1	7.5	6.4	30	7.4	25	31.8	7.6	2.5	11.22	0.2	6.2	22.2	33.1	6.3
15	M	76	L2	8	7	24	28.9	24.1	8.3	12.7	3.5	20.3	1.66	28.2	24.3	8.4	12.6
16	F	90	L1	7.5	20.3	11.48	14	25.6	10.4	7.2	3	20.5	1.88	10.9	19.8	8.6	8.8
17	F	78	L3	8.5	21	20.04	17	28	19	4.8	2	26.6	5.39	14	28	16	3
18	F	82	L1	8.5	24.8	15.44	27.2	33.3	7.1	1.2	3	20.15	5.66	27.4	33.3	7.3	1.3
19	F	79	T12	8	14.17	26.12	21.5	30	11	5.6	2	18.72	2.2	20.3	29.6	11.2	5.5
20	F	85	L1	9	9.6	28.64	16.7	34.3	3.02	1	1	17	1.88	15.8	28.9	5.8	1.2
21	F	71	L3	8	21.8	26.59	27.5	30.6	7.2	1.1	1.5	24.95	2.93	27.5	30.5	7.3	1.1
22	F	82	L3	8	13.75	23.9	15	30	14.3	1.6	1	17.75	1.91	14.8	29.6	13.2	1.7
23	F	78	L2	9	20.65	17.25	27.6	36.2	4.3	4.9	2	30.63	2.26	27.4	35.9	4.4	4.8
24	F	84	T10	8	19.25	26.5	14.7	17.8	15	7.9	1	20.2	0.49	14.5	17	15.2	8
Minima		71		6	6.32	11.48	7.4	17.8	0.45	0	1	11.22	0.2	6.2	17	2.4	0.3
Maxima		90		9	33.33	30	28.9	36.3	31.8	12.7	3.5	35.62	9.53	28.8	36.9	33.1	12.6
Mean		79.3		7.95	15.54	21.58	21.69	28.33	9.32	4.68	2.08	21.36	2.75	19.95	27.35	9.87	4.84
SD		7		0.61	7.22	4.35	5.77	4.08	6.71	3.7	0.72	6.24	2.72	6.43	4.37	6.36	3.17

*VAS* = visual analogue scale; *AH* = anterior height of the fractured vertebra; *KA* = kyphotic angle of the fractured vertebra; *AH* above = anterior height above the fractured vertebra; *AH* below = anterior height below the fractured vertebra; *SD* = standard deviation.

The mean preoperative VAS pain score was 8.9 ± 0.7 in Group I and 7.96 ± 0.61 in Group II. The mean preoperative AH of the fractured vertebra was 12.44 ± 6.22 mm in Group I and 15.54 ± 7.22 mm in Group II. The mean preoperative KA of the fractured vertebra was 18.17° ± 8.61° in Group I and 21.58° ± 4.35° in Group II. The mean preoperative AH above the fractured vertebra was 25.86 ± 5.4 mm in Group I and 21.69± 5.77 mm in Group II. The mean preoperative AH below the fractured vertebra was 26.08 ± 6.18 mm in Group I and 28.33 ± 4.08 mm in Group II. The mean preoperative KA above the fractured vertebra was 4.76° ± 5.73° in Group I and 9.32° ± 6.71° in Group II. The mean preoperative KA below the fractured vertebra was 5.11° ± 6.65° in Group I and 4.68° ± 3.7° in Group II.

The mean postoperative VAS pain score was 1.5 ± 1.3 in Group I and 2.08 ± 0.72 in Group II. The mean postoperative AH of the fractured vertebra was 19.57 ± 3.89 mm in Group I and 21.36 ± 6.24 mm in Group II. The mean postoperative KA of the fractured vertebra was 9.67° ± 5.18° in Group I and 2.75° ± 2.72° in Group II. The mean postoperative AH above the fractured vertebra was 25.67 ± 5.29 mm in Group I and 19.95 ± 6.43 mm in Group II. The mean postoperative AH below the fractured vertebra was 26.2 ± 6.2 mm in Group I and 27.35 ± 4.37 mm in Group II. The mean postoperative KA above the fractured vertebra was 4.84° ± 5.67° in Group I and 9.87° ± 6.36° in Group II. The mean postoperative KA below the fractured vertebra was 4.55° ± 6.61° in Group I and 4.84° ± 3.17° in Group II.

Preoperatively, there was no significant difference between the groups in terms of the symptomatic level (p = 0.845), VAS score (p = 0.539), KA of the fractured vertebra (p = 0.43) or AH above the fractured vertebra (p = 0.196). On average, Group II patients (79.3 years) were older than Group I patients (73.6 years) (p = 0.008). Before the operation, the AH of the fractured vertebra and AH below the fractured vertebra values were lower in Group I patients as compared to Group II patients (p = 0.004 and p < 0.001, respectively). Preoperative measurements of KA above the fractured vertebra were larger in Group II as compared to Group I (p = 0.009). KA below the fractured vertebra was higher in Group I than Group II patients (p < 0.001) before the operation. Notably, the data were adjusting for preexisting differences in terms of gender, fracture level, age, and preoperative clinical data using the analysis of covariance for two nonequivalent groups.

The postoperative measurements were also compared between groups. There was no significant difference between the groups in terms of VAS score (p = 0.198), AH of the fractured vertebra (p = 0.775), AH above the fractured vertebra (p = 0.64) or KA below the fractured vertebra (p = 0.266). However, the AH below the fractured vertebra was significantly higher and the KA above the fractured vertebra was significantly smaller in Group I than in Group II (p = 0.029 and p = 0.008, respectively). The KA of the fractured vertebra was significantly smaller in Group II than in Group I (p < 0.001).

No case of I-VEP fatigue, anterior or posterior loss of I-VEP, pulmonary embolism, cement extravasation, or infection was reported. However, one patient (Patient 8 in Group I) experienced operation-related complications. This patient experienced right-leg weakness soon after the operation and had recovered completely by the 6-week follow-up.

**Figure 6:**
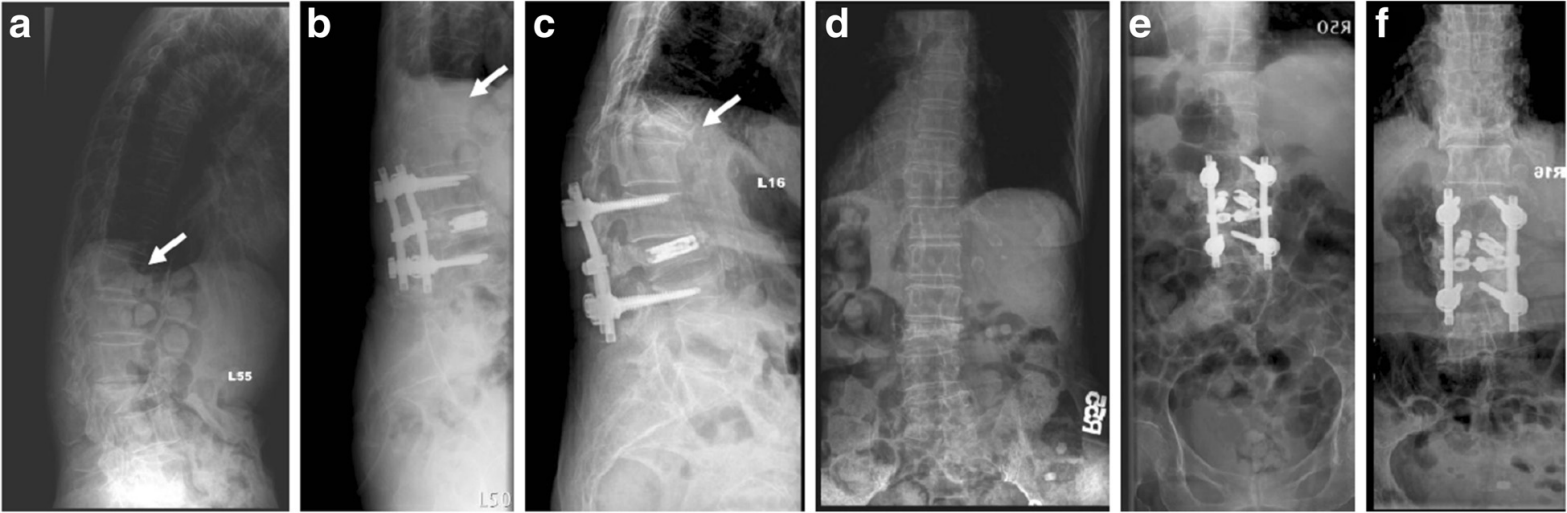
**a The lateral view of Patient 1 in Group I shows an L3 vertebral compression fracture and an intact T12 before the operation. b** T12 was further collapsed at the three-month follow-up. **c** The recent T12 fracture remains at the one-year follow-up. **d** Anteroposterior view, preoperative radiograph. **e** Anteroposterior view, three-month follow-up. **f** Anteroposterior view, one-year follow-up.

Patient 1 in Group I (an 85-year-old woman), who was treated with short-segment fixation with I-VEP due to VCF of L3, suffered a further collapse at T12 three months after the operation. The anterior vertebral height of T12 was decreased from 27.5 mm before the operation to 8.6 mm at 3 months postoperatively; this value remained the same at the 1-year follow-up examination (Figure 6).

**Figure 7:**
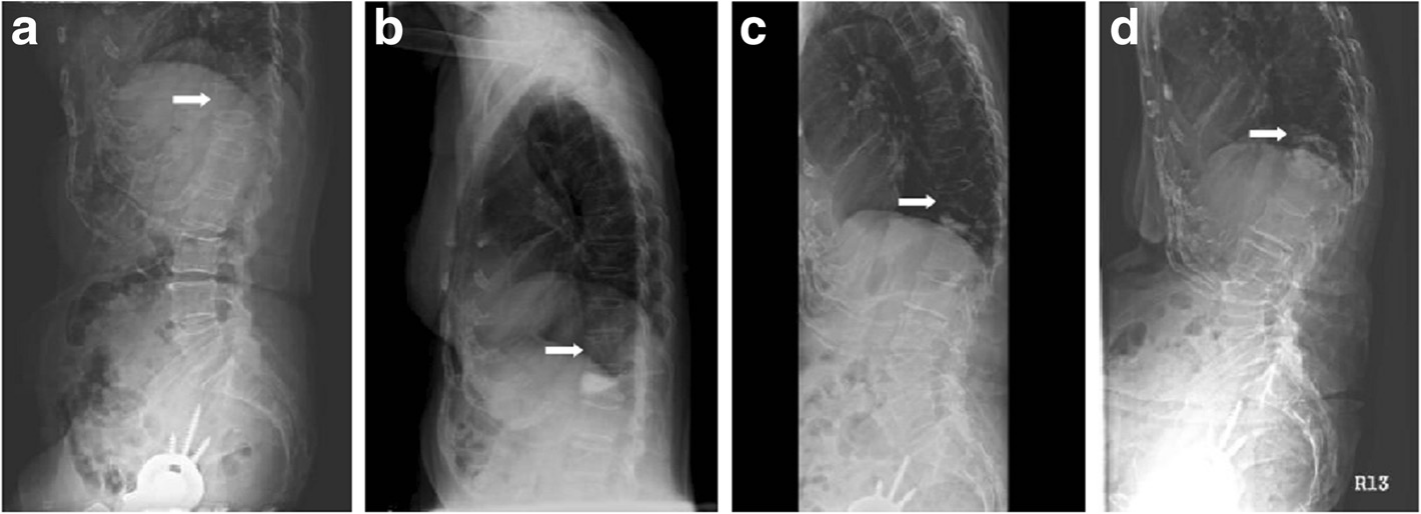
**a The lateral view of Patient 1 in Group II shows an T12 vertebral compression fracture and an intact T11 before the operation. b** T11 was intact initially after kyphoplasty for T12. **c** Adjacent T11 fracture was noted at the three-month follow-up. **d** The domino effect of T11 was further observed till the one-year follow-up.

Patient 1 in Group II (an 84-year-old woman), who was treated with KP due to VCF of T12, suffered a further collapse at T11 three months after the operation. The anterior vertebral height of T11 was decreased from 24.9 mm before the operation to 12.1 mm at 3 months postoperatively and 9.8 mm at the 1-year follow-up examination (Figure 7).

## Discussion

There are several alternatives for the treatment of patients with VCF, which vary in terms of the augmentation material used for restoration and maintenance within the collapsed vertebra. The most widely used procedure, which is considered as the gold standard in the field, is vertebroplasty (or KP with a PMMA alternative). The complications of this approach can include the symptomatic extravasation of cement and a significantly increased risk of vertebral collapse at the adjacent, non-augmented level [17]. PMMA added to the osteoporotic cancellous bone augments quite stiffly, lacks osseointegration and has limited biocompatibility, which may result in the collapse of adjacent vertebra [18].

**Figure 8:**
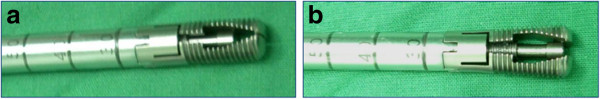
**a The I-VEP is in resting status. b** The I-VEP is in expanding status.

**Figure 9:**
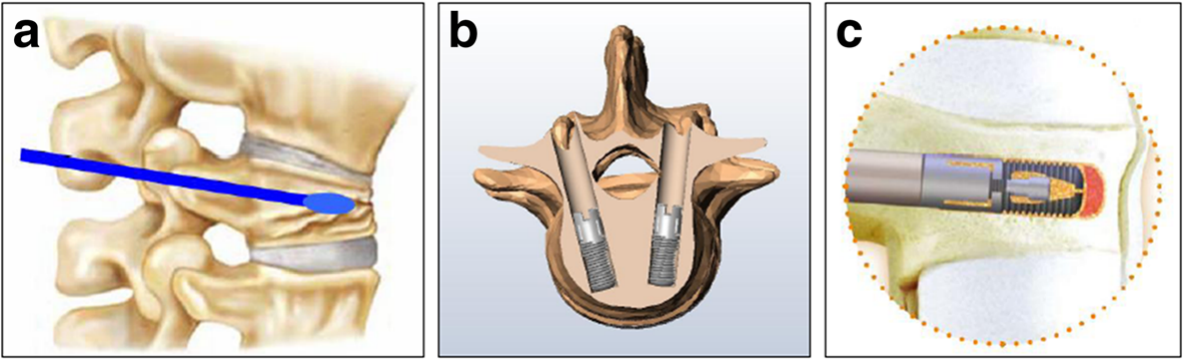
**Illustration of I-VEP placement. a** Detection of the pedicle tract, insertion of the probe to the collapse area and entrance into the center of the vertebra in the sagittal plane. **b** Convergent insertion of I-VEP into the vertebrae 2 mm away from the anterior cortex after dilatation of the tract and creation of void space. **c** Pack both inside and out with bone chips in the hollow I-VEP.

The I-VEP (30 mm in length and 10 mm in unexpanded diameter) was designed as a hollow threaded cylinder and an inner screw within the conical cavity (Figure 8). After the I-VEP was filled up with autologous cancellous bone chips, the implant was then screwed into the vertebra 2 mm away from the anterior cortex through the same pedicle tract using a holding handle. I-VEP can be expanded by 3°–4° after fastening the inner screw into the conical cavity through the holding handle using a customized screwdriver. The proper position and the expanded status of the implant was checked by serial C-arm fluoroscopic surveillance to make sure it was within the vertebra and fully expanded. I-VEP was disconnected from the holding handle after inspection of good position of the implant in the central vertebra and being fully expanded with well restoration of the vertebral height (Figure 9). The reversal procedures would be performed if the I-VEP was needed for removal.

The biological augmentation of I-VEP is used to reconstruct the vertebra through internal mechanical support and also by encouraging bony fusion. In addition to being enveloped by bone chips, the I-VEP is made of titanium alloy, which is known for its excellent biocompatibility. Just like the anterior expandable strut cage replacement or the expandable cage, the I-VEP can be filled up with bone chips, which expand after settling [19]. However, the I-VEP was implanted through the posterior approach and without corporectomy. Omitting corporectomy could diminish the surgical risk of neurovascular damage and blood loss. Furthermore, preservation of the end plates prevents subsidence of the I-VEP into the adjacent segments.

Patient 1 in Group I suffered a further collapse at T12 three months after short-segment fixation with I-VEP for L3 VCF. However, it is difficult to differentiate a novel instance of VCF from the natural process of aging or a complication related to short-segment fixation with I-VEP; T12 was not just above the fracture level and not defined as an adjacent cephalic vertebral fracture in this case after all. Patient 1 in Group II suffered a further collapse at T11 after KP for T12 VCF till the 1-year follow-up examination. T11 was just above the fracture level in this case. It may be the domino effect of an incurring further VCF at the adjacent segment from the natural process of aging or a complication related to KP.

The AH of the fractured vertebra and AH above the fractured vertebra were similar in the preoperative evaluation and at the final follow-up. A higher AH below the fractured vertebra and smaller KA above the fractured vertebra were noted in Group I at the 1-year follow-up examination, which shows that adjacent collapse was less frequent among the patients who underwent short-segment fixation with I-VEP as compared to those treated with KP with PMMA.

Pedicle instrumentation with fusion for the treatment of thoracolumbar fractures might not be as effective in short segments as in long segments, which would manifest as a higher risk of loss of reduction or implant failure [20-22]. Osteoporosis renders bone fragile, which can lead to treatment failure. The use of intracorporeal devices in addition to bone grafting for internal support to maintain body height and support cancellous bone regeneration has been reported and provides a new option for treating VCF [23,24]. The combination of short-segment fixation with I-VEP represents a less invasive procedure that allows for intracorporeal augmentation. It has been reported that KP can restore vertebral height from 3–5 mm and reduce kyphotic deformity by 3°–14° [15,25]. The augmented I-VEP was 14 mm in expanded diameter, with a diameter of only 3°–4° as an expanded trumpet-shaped cylinder (Figure 9). It was not unexpected that KP maintained the sagittal correction of kyphotic deformity more effectively than short-segment fixation with I-VEP, but both techniques were similarly effective in restoring vertebral height.

Due to the use of serial dilation and C-arm fluoroscopic surveillance, this study involved a low rate of serious neurological complications after placement of the solid I-VEP. The rate of complications was higher in patients who were injected with liquid PMMA by KP [26]. In older individuals with VCF, the collapsed vertebral body was restored and maintained with less kyphotic deformity with the use of KP. Short-segment fixation with I-VEP is inferior to KP with respect to the maintenance of local kyphotic deformity. However, the former technique results in less adjacent collapse as measured by the AH below and the KA above the fractured vertebra. Short-segment fixation with I-VEP appears to be comparable to the gold-standard procedures for VCF in terms of alleviating pain, restoring the height of the injured vertebra and the vertebra immediately superior, and maintaining the level of kyphotic deformity one level below the injury. Finally, short-segment fixation with I-VEP was superior to other techniques in its ability to keep adjacent segments intact, which prevented the domino effect that results in an osteoporotic kyphotic spine [27].

Vertebral body height was only measured in the anterior cortex of the fractured vertebra; a more complete analysis would have included measurements of the anterior, central and posterior cortices. The simplicity of our analysis, which considered only the VAS pain assessment and two radiological parameters (AH and KA of the fractured vertebra), allowed for direct evaluation of the most relevant experimental parameters. Measurements of posterior height of the fractured vertebra in the KP group would have obfuscated the central question of our research, because the short-fusion group would have yielded similar AH of the fractured vertebra but larger KA of the fractured vertebra. Similarly, posterior height of the fractured vertebra would be reduced at the cephalic adjacent vertebra in the short fusion group due to the smaller KA of the fractured vertebra, despite the similarity AH of the fractured vertebra. Along the same lines, posterior height of the caudal adjacent fractured vertebra would have been reduced in the KP group due to values of KA of the fractured vertebra that were similar to those of the caudal adjacent vertebra, despite larger AH of the fractured vertebra in the short-fusion group. The results of various surgical techniques should be further evaluated and analyzed with respect to posterior height of the fractured vertebra.

There were some limitations in our series. First, this study was retrospective and different surgeons performed the two procedures. The current findings, therefore, need to be further validated with larger samples in a multicenter comparative study. Second, our results may not bear comparison with those reported previously. The results of this study were based on a minimum of one year’s follow-up. Previous studies have examined patients after at least five years of follow-up. Those studies examined a younger patient population (average age, 45.2 and 48.8 years, respectively) [28,29] than investigated in this report. Five-year follow-up would have been extremely challenging in our study population. Another point of departure lies in the fact that previous studies examined long segmental fusion in young adult patients with scoliosis, whereas this investigation focused explored short-segment fixation with I-VEP for osteoporotic vertebral fracture in elderly patients.

## Conclusion

The percutaneous balloon kyphoplasty with PMMA is recommended for the relief of pain among extremely senile patients with complicated comorbid diseases. The results presented here show that, after adjustment for gender, fracture level and age, kyphoplasty was superior to other surgical techniques in restoring the kyphotic deformity of collapsed vertebral bodies in VCF patients. The use of short-segment fixation with I-VEP to preserve AH below and KA above the fractured vertebra kept the adjacent segments intact, which may offer an alternative treatment for patients with VCF. This approach offers a comparable level of pain relief, maintains the integrity of adjacent structures, and reduces the likelihood of a domino effect up to one year postoperatively. However, the immediate and early pain relief achieved with kyphoplasty may be more meaningful than the long-term prevention of a domino effect in extremely senile patients with comorbidities. Further research about the biomechanical stability of the spine in this context and more long-term clinical data will be needed to definitively evaluate the role of the two techniques in the treatment of patients with osteoporotic vertebral compression fracture.

## Competing interests

We declare that we have no competing financial or non-financial interests in relation to this manuscript.

## Authors’ contributions

PQC took part in study conception and design, data acquisition, drafting of the article, and revision of the article. JYH took part in study conception and design, statistical analyses, interpretation of the results, and implementation of the article. CDW, TMW, HYC and CJF participated in study conception and design, as well as execution of the study. All authors approved the final version.

## Pre-publication history

The pre-publication history for this paper can be accessed here:

http://www.biomedcentral.com/1471-2474/14/75/prepub
